# Role of Transportome in the Gills of Chinese Mitten Crabs in Response to Salinity Change: A Meta-Analysis of RNA-Seq Datasets

**DOI:** 10.3390/biology10010039

**Published:** 2021-01-08

**Authors:** Adeel Malik, Chang-Bae Kim

**Affiliations:** 1 Institute of Intelligence Informatics Technology, Sangmyung University, Seoul 30316, Korea; 2 Department of Biotechnology, Sangmyung University, Seoul 30316, Korea

**Keywords:** Chinese mitten crab, *Eriocheir sinensis*, transportome, transporters, salinity, osmoregulation, transcriptome, RNA-Seq, meta-analysis, gills

## Abstract

**Simple Summary:**

*Eriocheir sinensis* is a freshwater crab and is considered as one of the most important cost-effective species for freshwater aquaculture. *E. sinensis* can grow in both freshwater and brackish waters. In order to adapt to this changing salinity, *E. sinensis* can regulate the osmotic concentration of its hemolymph. Additionally, studies have shown that gills are one of the most important tissues in osmoregulation. In this work, we performed the first meta-analysis of publicly available RNA-Seq datasets to identify differentially expressed genes in the gills under different salinity conditions. The results highlighted that many different types of transporters show altered expression because of salinity change. Some of these transporters may serve as novel or new biomarkers for osmoregulation. The findings of this work also suggest that cellular processes related to many morphological changes are also affected.

**Abstract:**

Chinese mitten crab (CMC) or *Eriocheir sinensis* is a strong osmoregulator that can keep rigorous cellular homeostasis. CMC can flourish in freshwater, as well as seawater, habitats and represents the most important species for freshwater aquaculture. Salt stress can have direct effects on several stages (e.g., reproduction, molting, growth, etc.) of the CMC life cycle. To get a better overview of the genes involved in the gills of CMC under different salinity conditions, we conducted an RNA-Seq meta-analysis on the transcriptomes of four publicly available datasets. The meta-analysis identified 405 differentially expressed transcripts (DETs), of which 40% were classified into various transporter classes, including accessory factors and primary active transporters as the major transport classes. A network analysis of the DETs revealed that adaptation to salinity is a highly regulated mechanism in which different functional modules play essential roles. To the best of our knowledge, this study is the first to conduct a transcriptome meta-analysis of gills from crab RNA-Seq datasets under salinity. Additionally, this study is also the first to focus on the differential expression of diverse transporters and channels (transportome) in CMC. Our meta-analysis opens new avenues for a better understanding of the osmoregulation mechanism and the selection of potential transporters associated with salinity change.

## 1. Introduction

Chinese mitten crab (CMC), also known as *Eriocheir sinensis*, is a freshwater crab native to China and found as an invasive species in Europe and the United States of America (USA) [1]. This crab is one of the most essential cost-effective species for freshwater aquaculture in China, the Eastern Pacific coast, and the Korean Peninsula [2]. CMC is one of the unique crustaceans in a sense that they require two differing environmental conditions to complete their life cycle. As an adult, CMC spends its life in freshwater and later moves to a brackish (saline) water for reproduction [3]. Thus, CMC can regulate the osmotic concentration of its hemolymph so that it can better adjust to the new saline environment [4]. Therefore, these unique features make CMC a model organism among crustaceans to study the mechanism of osmoregulation under varying salinity conditions. All organisms exhibit adaptation to extracellular salinity within clearly defined ranges [5]. However, the mechanism is quite complex, and therefore, to better understand the response of these organisms towards salinity stress requires not only the knowledge of its molecular components but a thorough understanding of the complete biological system. To maintain the osmotic homeostasis during salinity stress, suitable signal transduction pathways are triggered in the aquatic animals [6,7,8]. However, there is limited knowledge regarding such signal transduction events that regulate osmoregulation in an aquatic organism [7]. The regulation of osmotic pressure and ionic balance in response to a change in salinity is an energy-dependent process in which lots of energy is utilized [9]. The significance of such energy metabolism-related pathways has been highlighted in previous studies [9,10]. One of the key groups of proteins that maintain osmotic pressure at the expense of huge quantities of energy are various ion transporters and ion transport channels [11]. All these membrane transporters and channels are collectively known as the “transportome”, which regulates the movement of various ions, nutrients, and drugs across biological membranes [12]. Sodium/potassium-transporting ATPase (Na^+^/K^+^-ATPase) of the gill epithelium is one of the essential ion transporters for hyperosmotic crustaceans and has been implicated in osmoregulation, as well as ion regulation [13]. Transporters such as Na^+^-K^+^-2Cl cotransporter-1 (*NKCC1*) that belongs to the chloride–cation cotransporters gene family [14] are involved in the secretion of Cl^−^ from the body of crustaceans to accomplish salt and water retention [9]. Similarly, V-type ATPase is one of the essential components of sodium uptake in the gills [15] and, therefore, provides a driving force for sodium to enter into gill epithelial cells via an epithelial sodium channel [13].

Some of these transporters have been highlighted in transcriptome-based studies of CMC during salinity stress. Specifically, such RNA-Seq-based studies in the gills of *E. sinensis* have mainly focused on the key transporter genes, such as Na^+^/K^+^-ATPase, *NKCC1*, and V-type ATPase [3,9,16]. However, in addition to these well-known biomarker genes, there are several other ion transport enzymes that are involved in ion transport [17]. Moreover, the uptake of ions by branchia in osmoregulating crustaceans is accomplished by the regulated activity of an array of transport proteins and transport-related enzymes [18]. Currently, more than 1500 transporter families have been described based on their functional and phylogenetic information [19]. Broadly, all these families are grouped into seven major classes in this transport classification (TC) system and include (i) channels or pores (TC 1), (ii) electrochemical potential-driven transporters (TC 2), (iii) primary active transporters (TC 3), (iv) group translocators (TC 4), (v) transmembrane electron carriers (TC 5), (vi) accessory factors involved in transport (TC 8), and (vii) incompletely characterized transport systems (TC 9). Many of these transporters are multi-subunit channels encoded by several genes [20], of which only a limited number of such subunits are highlighted. Therefore, in order to get a broader understanding of the diverse types of transporters involved in osmotic regulation in the gills of CMC (which lacked in the previous RNA-Seq-based studies of *E. sinensis*), we carried out a meta-analysis of four independent RNA-Seq studies. RNA-Seq has become the method of choice for differential expression analyses; however, due to their high costs, limited number of biological replicates are common in studies, which, in turn, leads to a low detection of differentially expressed genes (DEGs) [21]. Therefore, a meta-analysis of several independent studies focused on a specific biological question offers a useful way to overcome the limited detection power of independent studies. Meta-analysis approaches are a set of statistical techniques that merge multiple and independent studies to get a single common and significant outcome [22]. In a meta-analysis, transcriptome data from multiple individual studies can be analyzed in order to define common molecular signatures, improve reproducibility, or find more reliable biomarkers [23,24]. To the best of our knowledge, this study is the first ever RNA-Seq meta-analysis to investigate the in-depth account of various transporters implicated in salinity stress in *E. sinensis*.

## 2. Materials and Methods

### 2.1. Datasets

The National Center for Biotechnology Information (NCBI) BioProject (https://www.ncbi.nlm.nih.gov/bioproject) database was queried to search for RNA-Seq studies in response to salinity changes in *E. sinensis*. Studies that focused on a change in salinity from freshwater to higher salinity using tissue samples from gills were selected only. RNA-Seq reads (fastq files) for the selected samples from each study (Table 1) were retrieved from the NCBI’s sequence read archive (SRA: https://trace.ncbi.nlm.nih.gov/Traces/sra/) database by using the fastq-dump program of the SRA Toolkit (http://ncbi.github.io/sra-tools/).

### 2.2. De Novo Transcriptome Assembly

Prior to assembly, Trimmomatic [25] within the Trinity v.2.10.0 [26] pipeline was used to filter the reads by removing the adapter sequences, and then, the reads were scanned with a 4-base-wide sliding window, cutting when the average quality per base dropped below 20. Furthermore, the reads were removed if their lengths dropped below 50% of the original read length. De novo transcriptome assembly was performed on the resulting quality filtered reads by using the docker image of Trinity v.2.10.0. Each dataset was assembled separately.

### 2.3. Assembly Statistics and Completeness

Assembly statistics were calculated by using the TrinityStats.pl script provided within the Trinity pipeline. The percentage of reads mapping back to the assembly was computed with Bowtie2 [27]. BUSCO v.4.0.6 [28] was used to assess the transcriptome completeness of all datasets against the arthropoda_odb10 lineage. To quantify the transcript abundance, we used the ultra-fast alignment-free method kallisto [29] bundled within the docker image of Trinity.

### 2.4. Functional Annotation of the Transcripts and Enrichment Analysis

Potential coding regions and open reading frames (ORFs) were predicted with the TransDecoder [26] pipeline using the default parameters. The resultant protein sequences were then scanned against the UniProtKB/Swiss-Prot [30] database using BLASTp [31]. In contrast, HMMER v.3.1b2 [32] was used to identify the protein domains against the Pfam [33] database. Additionally, we also scanned the protein sequences from each dataset against the amino acid sequences (Crabdb) previously identified from the draft crab genome [34]. Orthologous groups were identified by using the online functional annotation tool eggNOG-mapper [35]. The classification of differentially expressed transcripts (DETs) into various transporter classes was performed by a BLASTp-based search against the Transport Classification Database (TCDB) [19]. Enrichment of gene ontology (GO) terms and KEGG pathways of the DETs were performed using the ShinyGO v.0.61 [36] and KOBAS v.3.0 [37] web-based tools. Enriched categories with a corrected *p*-value < 0.05 cutoff were selected only.

### 2.5. Differential Expression and Transcriptome Meta-Analysis

Differential expression (DE) analysis for individual studies was performed at the isoform level using the edgeR [38] package within the Trinity pipeline. The output of the DE analysis from all datasets was further employed for the meta-analysis, which was carried out by using the metaRNASeq [39] package. metaRNASeq uses Fisher’s combined probability test [40] for meta-analyses and has been used in several studies [41,42,43]. As the name suggests, this method combines the *p*-values for each gene from individual studies using the following formula [39,40]:

$$F_{g}=-2\overset{s}{\underset{s=1}{\sum }}ln\left(p_{gs}\right)$$

Here, *p_gs_* corresponds to the raw *p*-value calculated for a gene (*g*) in a differential analysis for the study (*s*). With independent *p*-values, *F_g_* has a *χ*^2^ distribution with 2S degrees of freedom. Smaller *p*-values result in larger *F_g_*, and therefore, the null hypothesis is rejected [43]. *p*-values were adjusted for the Benjamini–Hochberg false discovery rate (FDR), and a corrected *p*-value < 0.05 was considered as statistically significant [44]. Transcripts (genes) that exhibited an FDR of <0.05 and an average fold change (FC) of ≥2 (log_2_FC ≤ −1 or log_2_FC ≥ 1) were considered as DETs in the meta-analysis. Heatmap and correlation analyses were conducted by using the pheatmap (https://cran.r-project.org/package=pheatmap) and Hmisc (https://cran.r-project.org/package=Hmisc) packages, respectively, within the R (https://www.r-project.org/) programming environment.

### 2.6. Network Analysis and Community Detection

The amino acid sequences of the DETs were mapped to the STRING v.11.0 [45] database (STRING-DB) to predict the interactions between these sequences using *Drosophila melanogaster* (fruit fly) as the reference organism. The interaction network of the DETs was visualized and further analyzed with Cytoscape v.3.8.0 [46]. Node degree distribution and community structure were estimated by using the NetworkAnalyzer [47] and GLay [48] plugins of Cytoscape, respectively.

## 3. Results

### 3.1. Datasets and De Novo Transcriptome Assembly

Based on the search criteria (see Methods), four RNA-Seq datasets were retrieved for this study. Although all these studies used freshwater and high salinity samples, the concentration of salt in higher salinity groups varied. For example, the salinity in two of these studies was 30% [3,10], whereas the other two used 20-ppt and 25-ppt saline water [9,16], respectively. All the selected datasets were previously published, and among them, three were pair ends, and one study represented single-end reads. Table 1 summarizes these datasets and provides statistics on reads from each individual dataset. We de novo assembled each of these RNA-Seq datasets separately. Furthermore, to assess the read composition of our assembly, we generated statistics on all reads that map to our assembled transcripts. We observed that the number of reads that mapped back to the assembly ranged between 94% to 97% (Table S1). These numbers are above 80% and signify a good quality assembly [26]. The average contig length ranged between 630.8 and 889.08 for DS4 and DS3, respectively. Similarly, the N50 value for DS4 was lower (974), whereas DS3 had the largest (1526) N50 value. The completeness of the assembled transcripts with BUSCO showed the percentage of orthologs among the Arthropods represented in our assembled CMC transcriptomes. Specifically, of the 1013 BUSCO groups searched, a high percentage (72–94%) of complete orthologs belonging to the phylum Arthropods were detected for each dataset (Figure S1A). This includes both the complete single copy, as well as complete duplicates (putative paralogs or complete genes with multiple copies). For non-model organisms such as *E*. *sinensis*, BUSCO scores within the range of 50–95% are expected [28].

### 3.2. Identification of Coding Regions and Functional Annotation

TransDecoder identified 179,118, 279,120, 52,132, and 147,537 transcripts with the longest open reading frames (ORFs) for four CMC datasets (DS1, DS2, DS3, and DS4), respectively. Each of these ORFs were at least 100 amino acids long, and sequences shorter than 100 residues were excluded. These transcripts were then scanned against the UniProt, Crabdb, and Pfam databases to identify ORFs with homology to known proteins. Based on the BLASTp search, the percentage of sequences that mapped to the UniProt database ranged between 15.71% (DS2) and 27.89% (DS1). These numbers are consistent and slightly better in some cases as compared to the previous studies on this non-model organism [9,10,16]. Interestingly, the number of longest ORF sequences that mapped to Crabdb were also in similar ranges, e.g., between 15.83% (DS2) and 23.39% (DS3) (Figure S1B). These low numbers could be attributed to the fact that, at present, the number of annotated sequences in Crabdb is only around 7500, suggesting that many of the transcripts would have no known match in this database. Similarly, about 12–22% of transcripts with long ORFs had orthologs in the KEGG database. In contrast, the number of orthologs identified by eggNOG-mapper were higher as compared to KEGG (Figure S1B). Specific COG categories for each dataset are provided as a supplementary figure (Figure S2). Among all the databases, transcripts across all datasets showed higher annotations against the Pfam database. The results from the UniProt and Pfam searches were then integrated to further select the coding regions by using TransDecoder. The results indicated that the total number of protein coding transcripts that shared some similarity with the known sequences in the reference databases ranged between 43.39% (DS2) and 48% (DS3) (Figure S1C). This suggests that many of the CMC transcripts could be novel that are not present in these databases. Nevertheless, it may still be possible to identify a higher number of transcripts sharing similarity with known sequences using other large databases such as the NCBI nonredundant (NR) protein database [9,16]. It may be noted that the size of the NR database is huge, and performing similarity searches against such databases is computationally very expensive.

### 3.3. Meta-Analysis of CMC Gills Transcriptome

**Figure 1:**
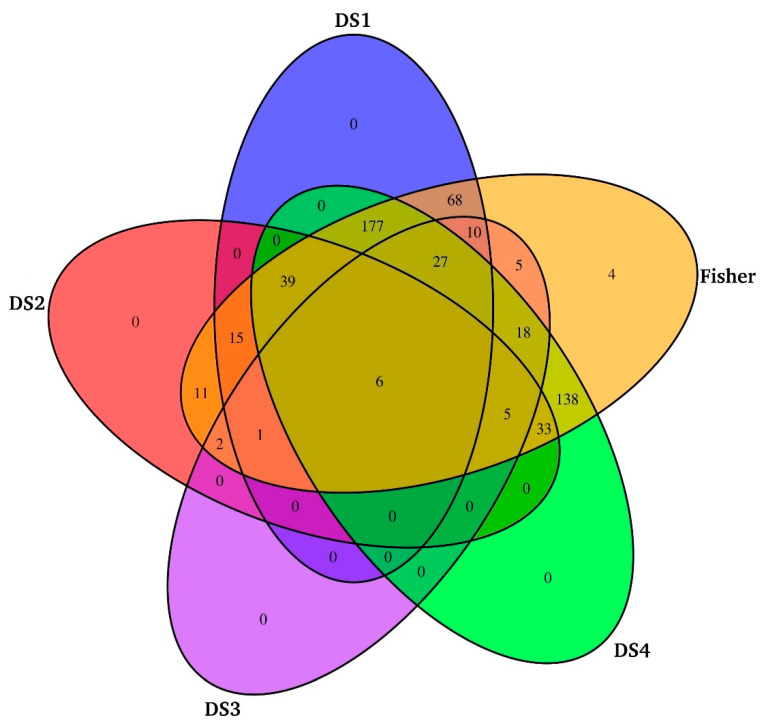
Venn diagram showing the number of differentially expressed transcripts identified from individual studies and meta-analysis based on the Fisher method.

Following the DE analysis of each individual dataset, we used the Fisher method and identified 3574 DETs across all four datasets. From this list, we first selected only those transcripts that exhibited an average fold change of ≥2 (log_2_FC ≤ −1 or log_2_FC ≥ 1) and an adjusted *p*-value of <0.05. This resulted in 1748 DETs, of which a large number of transcripts (~1300) were further excluded from the analysis because of their inconsistent or conflicting expression patterns. Such inconsistent transcripts were upregulated in some studies and exhibited downregulation in others. Therefore, only those transcripts that showed consistent expression in the four datasets were considered as DE (Figure 1).

**Figure 2:**
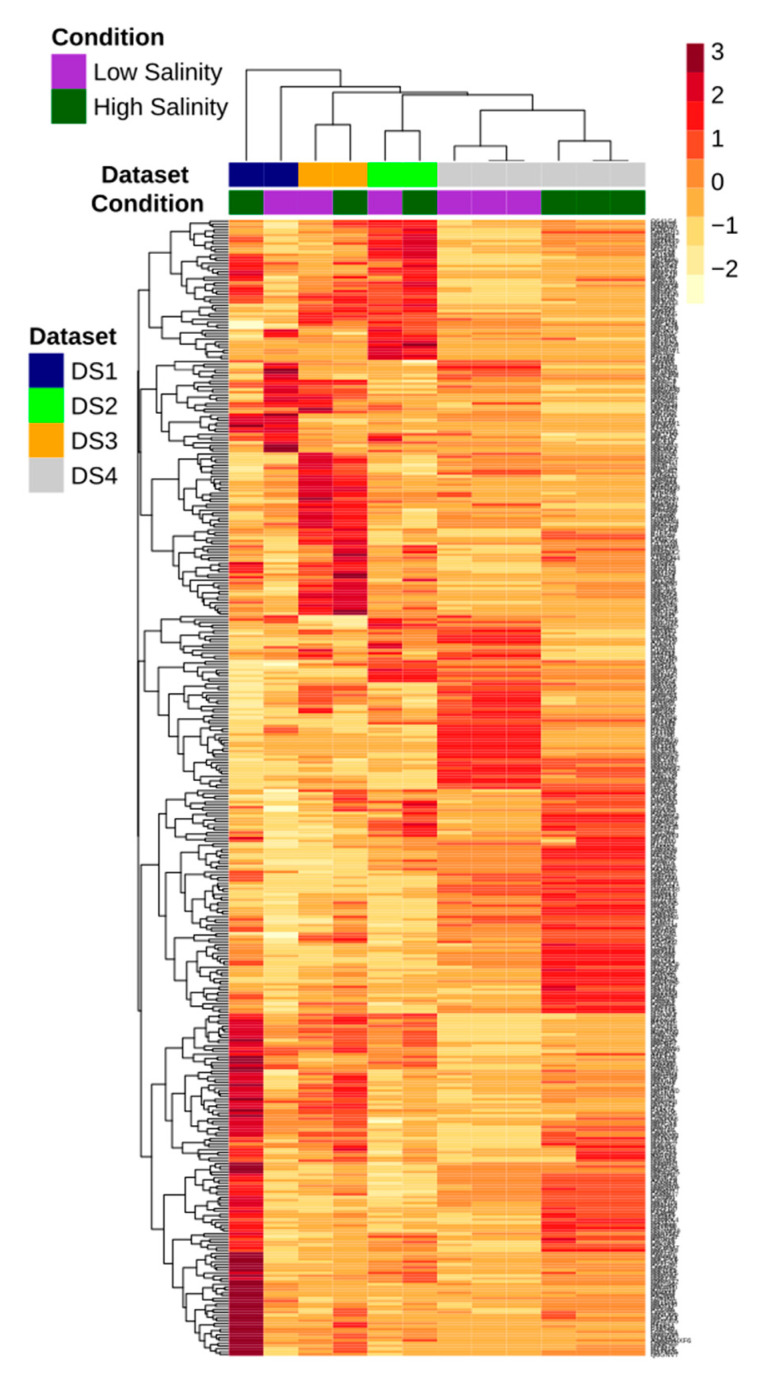
Heatmap showing differentially expressed transcripts identified by meta-analysis in the gills transcriptome of four different Chinese mitten crab (CMC) datasets under salinity. Only transcripts with an average fold change (FC) of ≥2.0 and a Benjamini-Hochberg adjusted *p*-value of <0.05 were considered to be differentially expressed.

For example, a transcript upregulated in one dataset was also upregulated in the other three datasets and vice versa. No gene that was identified as DE in the individual studies was lost in the meta-analysis. Although the meta-analysis identified four transcripts that were not detected as being DE in individual studies alone (Table S2), their average fold change was slightly lower (FC 1.5) as compared to the 2 FC criteria set in the current study. Hence, 405 DETs were finally retained for further downstream analysis (Figure 2).

**Figure 3:**
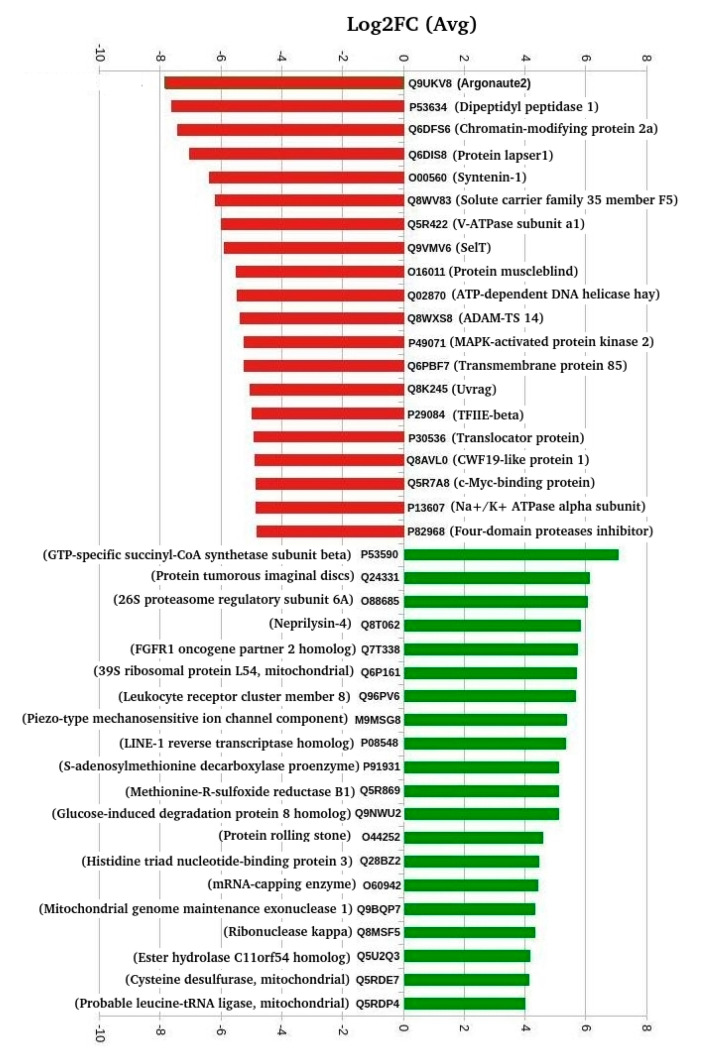
Top 20 up- and downregulated differentially expressed transcripts.

Of the 405 DETs, 272 were downregulated, whereas 133 were upregulated between freshwater and high salinity conditions. Figure 3 provides the overview of top 20 up- and downregulated transcripts, and the complete list of 405 transcripts with detailed annotations is provided as additional information in Table S3.

Moreover, several of the 405 DETs exhibited very high correlations (Table 2). From these data, we observed that the topmost downregulated transcript encodes for protein argonaute-2 (*AGO2*), whereas mitochondrial succinate–CoA ligase (GDP-forming) subunit beta (*SUCLG2*), also known as GTP-specific succinyl-CoA synthetase (G-SCS), was encoded by the topmost upregulated transcript. 

Among the list of 405 DETs, six (including one of the topmost upregulated, *SUCLG2*) were identified as DE across all four datasets, as well as by the Fisher method (Table 3).

### 3.4. GO and KEGG Enrichment Analyses of DETs

**Figure 4:**
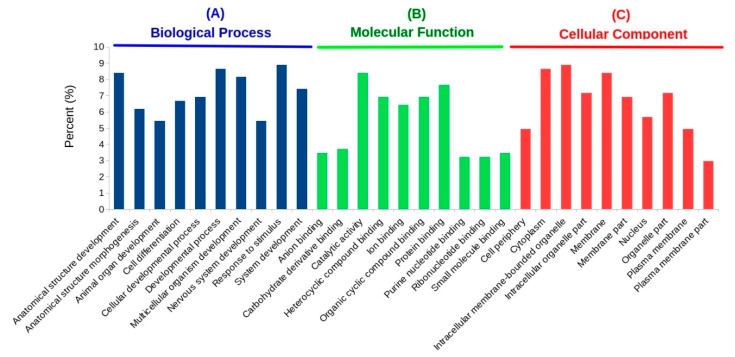
Gene ontology (GO) enrichment of differentially expressed transcripts identified by the meta-analysis. Only the top 10 processes for each GO category in terms of the biological process (BP), molecular function (MF), and cellular component (CC) are shown. A complete list of enriched terms is provided as Table S4.

Using the ShinyGO tool, enriched GO categories associated with DETs were identified in terms of the biological process (BP), molecular function (MF), and cellular component (CC). Based on this analysis, the GO term “response to stimulus” was the most enriched biological process, followed by several others related to development, such as anatomical structure development, multicellular organism development, and system development (Figure 4A and Table S4). Besides the enrichment of various developmental processes, an interesting observation was that out of 30 topmost enriched GO biological processes, at least six were related to the nervous system and included nervous system development, neuron differentiation, the generation of neurons, neurogenesis, neuron development, and neuron projection development (Table S4). Reports have suggested that, during osmotic changes, the neuroendocrine system may modulate ion transport in the gill epithelium through neurotransmitter signaling [9]. One of the key enzymes that was identified to be downregulated in high salinity as compared to freshwater conditions in the meta-analysis was the alpha subunit of sodium/potassium-transporting ATPase (Na^+^/K^+^-ATPase). This enzyme was found to be part of various enriched biological processes, such as the response to stimulus and nervous system development, in the meta-analysis.

We also identified DETs that encode five V-ATPase subunits (subunits a1, C, D1, H, and the proteolipid). The transcripts for all these subunits were upregulated by more than two FC, except in the case of subunit a1 (V-type proton ATPase 116-kDa subunit a1 encoded by the *ATP6V0A1* gene), which was downregulated. Among these, some of them, such as subunit H, were annotated to be involved in developmental processes (BP), whereas others (e.g., subunit D1) were involved in various molecular functions (MF), including nucleoside-triphosphatase activity, ATPase activity, etc. Additionally, the meta-analysis also identified subunits B, E, and G of the V1 cytoplasmic domain and d1 and e2 of the V0 membrane-bound domain of the V-ATPase complex to be differentially expressed. However, their expression patterns across all studies were conflicting.

One of the downregulated transcripts in the meta-analysis was identified to share similarity with integrin alpha-PS2 of *Drosophila* encoded by an inflated (*if*) gene. This transcript was identified in 27 out of 30 enriched GO biological processes, e.g., development, differentiation, morphogenesis, locomotion, and chemotaxis. The remaining three enriched processes in which integrin alpha-PS2 was not found were related to mRNA splicing. In *Drosophila*, such integrins are known to mediate the specific migration of tracheal cells in a given direction [49]. In contrast, this integrin was found in only two (protein binding and protein dimerization activity) enriched MF categories. Most of the enriched GO molecular functions were either related to the binding of various types of molecular compounds (e.g., nucleotide, purine, carbohydrate, small molecule, drug, etc.) or exhibited phosphatase/kinase activities (Figure 4B). The gene that was part of many enriched molecular functions encodes unconventional myosin IC (*Myo1C*), which has been implicated in many roles, including tension sensing and calcium-dependent cargo binding [50]. *Myo1C*, which was identified to be downregulated in the current study, belongs to a superfamily of actin-based motors that have roles in various cellular processes. Another unconventional myosin that was downregulated by more than four-fold was *Myo18a*, which plays an essential role in the organization and structure of the Golgi complex.

Among the GO cellular components, the most enriched were related to membranes (e.g., intracellular membrane-bounded organelles, the plasma membrane, the Golgi membrane, etc.); the cytoplasm; cell junction; and nucleus (Figure 4C). The proteins most frequently found in these categories include different subunits of V-type proton ATPase. 

The KEGG enrichment analysis identified 12 statistically significant pathways to be most important in the DETs. Most of these enriched pathways were related to metabolism, ECM-receptor interaction, and oxidative phosphorylation (Table 4). A total of 93 DETs were mapped to these pathways, of which 60 were downregulated, while the remaining 33 exhibited upregulation. One of the topmost downregulated transcripts encoded a charged multivesicular body protein 2a (*CHMP2a*), which belongs to the Snf family of small coil-coiled proteins. Such proteins are key components of the endosomal sorting required for transport complex III (*ESCRT-III*), which are essential for the formation of multivesicular bodies (MVBs) and the sorting of cargo proteins to MVBs [51]. V-ATPase subunit a1 was another topmost downregulated transcript identified to play essential roles in three different enriched KEGG pathways—namely, metabolic pathways and phagosome and oxidative phosphorylation. In contrast, the topmost upregulated gene, *SUCLG2*, was also implicated in KEGG metabolic pathways. The regulation of osmotic pressure via different transporters and channels is an energy-dependent process [11]. In a given tissue, the concentration of mitochondrial oxidative phosphorylation complexes is adjusted to the highest energy conversion requirements [52]. The activity of such mitochondrial complexes is regulated by tissue metabolic stress to sustain energy metabolism homeostasis. In addition to metabolic pathways, the roles of other pathways, such as the environmental information processing, signal transduction, and antioxidant pathways, were previously highlighted [3,9,10].

### 3.5. Interaction Network of DETs

STRING-DB was used to predict the interactions between these DETs using the common fruit fly (*Drosophila melanogaster*) as the source organism. Of these DETs, the amino acid sequences of only 57 (13 up- and 44 downregulated) transcripts were mapped to the fruit fly proteome, whereas an additional 231 proteins were predicted as their interacting partners. Therefore, the interaction network consisted of 288 nodes, each representing a gene, and 905 edges (interactions between nodes) (Figure S3). The node degree of each node in the network was calculated with the NetworkAnalyzer plugin of Cytoscape and ranged between 1 and 31. The topmost two nodes were considered as hubs, because they exhibited a node degree of ≥30. These nodes were identified to encode for a bifunctional glutamate/proline–tRNA ligase (*GluProRS*: node degree = 31) and a DNA-directed RNA polymerase II subunit RPB2 (*RpII140*: node degree = 30). Of these two, *GluProRS* was predicted by STRING-DB, whereas *RpII140* was one of the downregulated transcripts identified in the meta-analysis.

**Figure 5:**
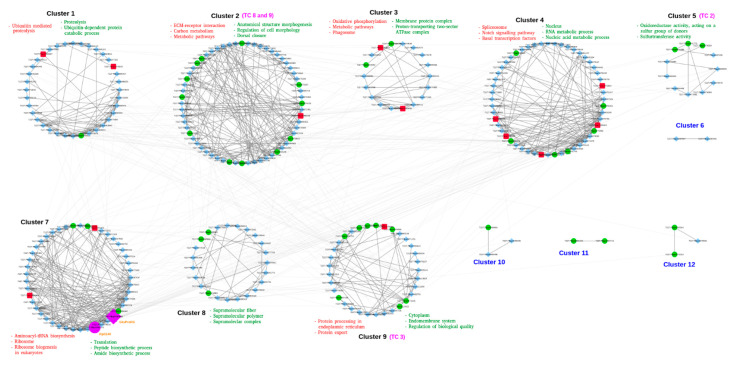
Community analysis of the interaction network. Red, green, and cyan nodes represent up- (square), downregulated (circle), and STRING-predicted (diamonds) genes, respectively. Nodes in purple indicate the hub genes labeled with orange text. The topmost enriched KEGG pathways (red text), GO terms (green text), and transporter class (pink text) are mentioned for each cluster. Clusters with <10 nodes (blue text) were excluded from the analysis.

There is a growing interest in the application of biological networks to address important biological phenomena [53]. Network-based studies offer a framework to get a broad overview of data that includes several interacting groups. This potential of a community-based network analysis allows researchers to investigate and compare a diverse set of proteins and their interactions within the context of modules or communities identified in the network [54]. Therefore, we attempted to identify the modular structure of the interaction network and identified that the network could be divided into 12 modules or communities (clusters) (Figure 5). However, only eight modules with 10 or more nodes (genes) were identified by using the GLay implementation of the fast-greedy algorithm [55] within the clusterMaker [56] plugin of Cytoscape. The algorithm finds clusters by repetitiously discarding edges from the network and then looks again for the linked nodes [57]. We performed an enrichment analysis for each of these eight individual clusters to identify enriched GO terms and KEGG pathways associated with these modules. The number of nodes in each of these clusters ranged between 11 (cluster 5) and 60 (cluster 2), respectively. The enrichment analysis of all the clusters suggested that each of these clusters was involved in performing specific functional roles. For example, most of the enriched GO processes in the largest cluster 2 were related to morphogenesis, development, or differentiation (Figure 5 and Table S5). In contrast, the smallest cluster 2 was enriched with processes related to oxidoreductase or sulfurtransferase activities, including others. Many transporters were also found in almost every cluster; however, only clusters 2, 5, and 9 were enriched with different transporter classes (Figure 5).

### 3.6. Transporters Implicated in Salinity Change

The above network analysis highlighted that almost all the clusters contained transporters; however, only clusters 2, 5, and 9 were enriched (*p* < 0.05) with different transporter classes (Figure 5). Moreover, most of the nodes in the network were proteins predicted as the interacting partners of a limited number of DETs that were mapped to STRING-DB. Therefore, to get a detailed account of the diversity of various types of transporters, we scanned the DETs against the TCDB using the BLASTp program. Although the role of transporters in the gills of CMC in response to salinity was suggested in previous studies, only a limited number and types of such transporters are highlighted. Most of these studies focused on the roles of only well-known markers, such as Na+/K+-ATPase, V-ATPases, and Cu2+ or Ca2+ transport ATPases [3,9]. Moreover, recognizing the fact that, at present, hundreds of transporter families have been described [19], focusing on only few marker genes does not provide the global overview of the role of the transportome in a salinity change. In this work, we identified that about 40% (162/405) of the DETs were classified as belonging to one or the other transporter class. These transporters are further described based on the most abundant classes or families identified in the DETs (Table 5).

#### 3.6.1. Accessory Factors Involved in Transport (TC 8)

**Figure 6:**
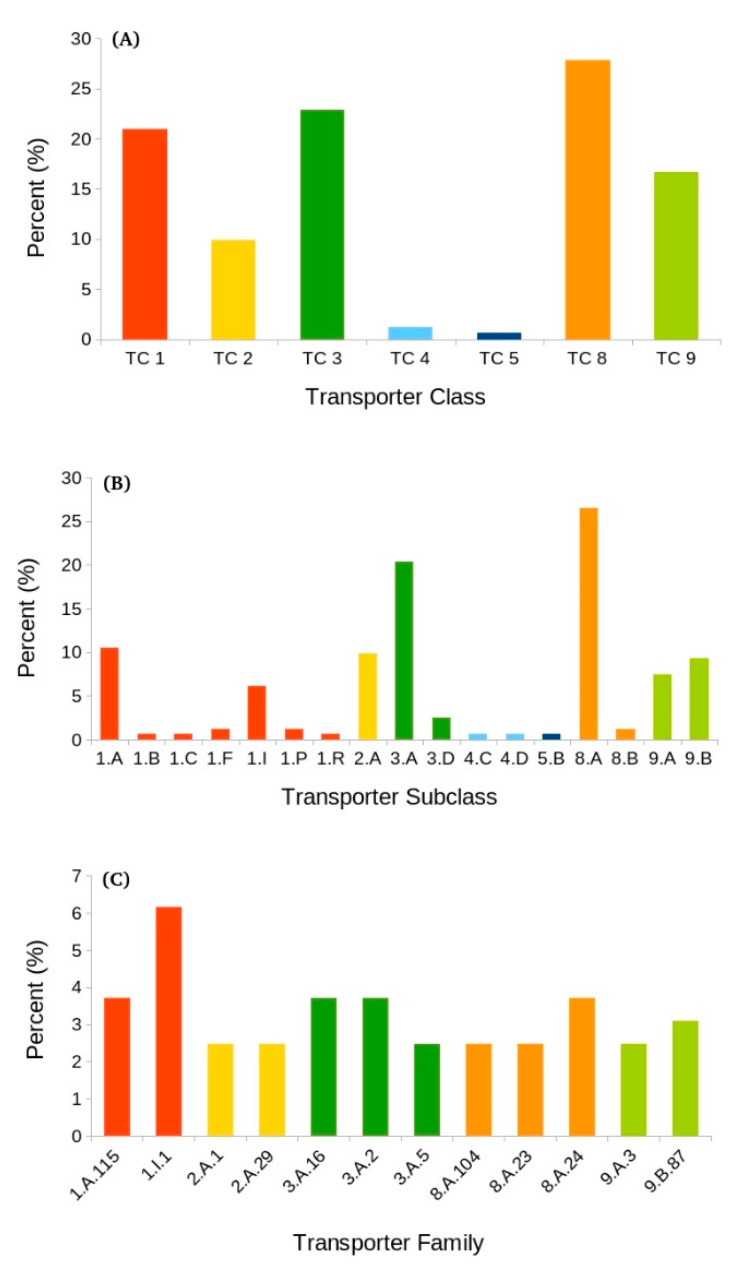
Classification of differentially expressed transcripts into various types of transporters at the (**A**) class, (**B**) subclass, and (**C**) family levels, as described in the Transporter Classification Database (TCDB) system.

This transporter class includes proteins that function with or are complexed to other known transport proteins. Of the 162 DE transporters, about 28% (45) represented class 8 transporters (Figure 6A). Almost all these transcripts belonged to subclass 8.A, except two, which were classified as subclass 8.B transporters (Table 5). Most of the transcripts (35/43) belonging to subclass 8.A were downregulated, whereas both of the 8.B transcripts were downregulated. Some of the most abundant TC 8 families are discussed below.

##### Auxiliary Transport Proteins (TC 8.A)

This subclass represents proteins that aid in transporting across one or more biological membranes but do not themselves take part in direct transport [19]. Such transporters consistently function cooperatively with well-established transport systems. Almost all the transcripts (43/45) assigned to TC 8 belonged to subclass 8.A, which also represented the most abundant transport subclass among all the identified subclasses (Figure 6B). These 43 transcripts were shared between 24 different families (Table S6).

Family TC 8.A.24: This was the second-most abundant transport family found in the meta-analysis (Figure 6C). Proteins belonging to this group represent the Ezrin/Radixin/Moesin-binding Phosphoprotein 50 (EBP50) family of transporters. EBP50, also known as Na^+^/H^+^ exchange regulatory cofactor (NHE-RF), is an adaptor protein that is known to manage a number of cell receptors and channels [58]. Several proteins of this family contain PDZ domains that are found in a wide variety of signaling proteins [59]. Six DETs were assigned to this family, of which five were downregulated, and only one transcript that encodes Salvador homolog 1 (*SAV1*) was upregulated. SAV1 consists of two WW domains, each consisting of about 33 amino acids. The name (WW) refers to two trademark tryptophan (W) residues placed 20–22 amino acids apart. Proteins with WW domains are involved in functions related to signaling or may play structural roles [60]. In addition to SAV1, the other seven upregulated transcripts representing class 8.A transporters were assigned to six other TC families (TC 8.A.28, 8.A.92, 8.A.96, 8.A.112, 8.A.128, and 8.A.131).

Family TC 8.A.23: Transcripts belonging to this group represent the basigin family of proteins that regulate transporters [61]. All the four DETs that were found in this family were downregulated and were annotated as LIM domain kinase 1, contactin, activated Cdc42 kinase-like, and tyrosine-protein kinase Abl. 

Family TC 8.A.104: This family comprises the catalytic subunits of AMP-activated protein kinases (*AMPK*), energy sensor protein kinases that are involved in regulating cellular energy metabolism [19]. All the four DET transcripts that fell within this family were downregulated by more than four-fold and represented cAMP-dependent protein kinase catalytic subunit beta, ribosomal protein S6 kinase alpha-5, serine/threonine-protein kinase pim-3, and dual serine/threonine and tyrosine protein kinase. In mammals, as the ATP levels within the cells decrease, *AMPK* triggers energy-producing pathways and suppress the processes that consume energy [62].

##### Ribosomally Synthesized Protein/Peptide Toxins/Agonists that Target Channels and Carriers (TC 8.B)

In contrast to 8.A transporters, the only two DETs that represented subclass 8.B were downregulated and belonged to the sea anemone peptide toxin, class 1 (BgK) family of transporters (TC 8.B.14). Such toxins are known to block potassium channels [63].

#### 3.6.2. Primary Active Transporters (TC 3)

TC 3 was the second-most abundant transport class after TC 8 and constituted about 23% (37 DETs) of the total transporters identified in the DETs (Figure 6A). These transporters aid in the active transport of solutes against a concentration gradient that is derived by a primary source of energy in the form of chemical, electrical, or solar energy [19]. Although there are five different subclasses of primary active transporters in the TCDB, only subclasses 3.A (89%) and 3.D (11%) were identified (Table 5).

##### P-P-Bond Hydrolysis-Driven Transporters (TC 3.A)

Of the 33 DETs identified in this group, TC 3.A was the second-most abundant subclass (Figure 6B), representing 14 different families. However, the three major families were identified as the H^+^- or Na^+^-translocating F-type, V-type, and A-type ATPase (F-ATPase) superfamily (TC 3.A.2), the Endoplasmic Reticular Retrotranslocon (ER-RT or ERAD) family (TC 3.A.16), and the general secretory pathway (Sec) family (TC 3.A.5).

Family TC 3.A.2: Of the six DETs representing the TC 3.A.2 family, five depicted various V-ATPase subunits (subunits a1, C, D1, H, and proteolipid), as described previously (see above), whereas the sixth DET encodes ribonuclease kappa (*RNASEK*).

Family TC 3.A.16: In the case of the six transporters belonging to the ERAD family (TC 3.A.16.), DETs representing 26S proteasome regulatory subunit 6A, cell division cycle protein 48 homolog MJ1156, and putative deoxyribonuclease TATDN1 were upregulated, whereas E3 ubiquitin-protein ligase AMFR, ubiquitin-conjugating enzyme E2 C, and ER degradation-enhancing alpha-mannosidase-like protein 2 were downregulated. Initially, it was thought that the ERAD system was entirely involved in the degradation of misfolded and orphan secretory proteins; however, the system has broad implications in various cellular processes, such as protein folding and transport, the regulation of metabolism, immune response, and ubiquitin-proteasome-dependent degradation [64].

Family TC 3.A.5: In addition to the TC families 3.A.2 and 3.A.16, the third topmost family in which all the four mapped DETs were downregulated by more than two-fold was TC 3.A.5 (the general secretory pathway (Sec) family). These 3.A.5 members were identified as calpain-3, signal recognition particle 54-kDa protein, transport protein Sec31a, and putative u5 small nuclear ribonucleoprotein 200-kDa helicase. Protein complexes belonging to the Sec family are found in both the prokaryotes, as well as eukaryotes. The Sec system essentially consists of two major components: (a) three integral inner membrane proteins, SecYEG, and (b) the ATPase motor protein SecA and represents one of the major ways by which protein export or membrane integration occurs in bacteria [65].

##### Oxidoreduction-Driven Transporters (TC 3.D)

Only four DETs distributed between two families—namely, the H^+^ or Na^+^-translocating NADH dehydrogenase (NDH) family (TC 3.D.1) and the proton-translocating cytochrome oxidase (COX) superfamily (3.D.4)—were included in this subclass. All the four transcripts assigned to this subclass were upregulated by ≥two-fold.

Family TC 3.D.1: As the name suggests, NADH:ubiquinone oxidoreductases type I (NDH-Is) of bacterial, as well as of eukaryotic, mitochondria and chloroplast origin, coupled with electron transfer to the electrogenic transport of protons or Na^+^ [66,67]. Of the four DETs identified in the 3.D subclass, three were found in this family and represent NADH dehydrogenase (ubiquinone) iron-sulfur protein 8 (mitochondrial) (*NDUFS8*), NADH dehydrogenase (ubiquinone) 1 alpha subcomplex subunit 13 (*NDUFA13*), and probable NADH dehydrogenase (ubiquinone) 1 alpha subcomplex subunit 12 (*Y94H6A.8*), respectively. The vertebrate H+-translocating NADH dehydrogenase (NDH) complex consists of 45 subunits [68], and among these, *NDUFA13* is crucial for membrane potential formation and NADH assembly [69].

Family TC 3.D.4: Only one DET was found in this family and represents mitochondrial cytochrome c oxidase subunit 7A1 (*COX7A1*). These multi-subunit enzyme complexes reduce O_2_ to water and, consequently, pump four protons across the membrane [19].

#### 3.6.3. Channels/Pores (TC 1)

The third-most abundant class of transporters was channels/pores with about 34/162 (~21%) DE transporters found in this class (Figure 6A and Table 5). Transporters in this group have transmembrane channels mainly consisting of α-helical or β-strand-type spanners [19]. In the TCDB, at least 23 different subclasses of channels are recognized; however, only seven such categories were found in this study (Table S6).

##### α-Type Channels (TC 1.A)

In general, these transporters consist mostly of α-helical spanners, although they may also contain some β-strands [19]. Of all the TC 1 transporters, 17/34 (50%) were found in this subclass. Furthermore, 11 different types of TC 1.A families were identified in this category.

Family TC 1.A.115: This was the most abundant family among all α-type channel transporters, with six DETs found in this group, and is represented by the pore-forming NADPH-dependent 1-acyldihydroxyacetone phosphate reductase (*AYR1*) family. Transporters like *AYR1* form an NADPH-regulated channel in lipid bilayers, as well as in the outer mitochondrial membranes [70]. Of the six transporters belonging to TC 1.A.115, four were downregulated and represented dehydrogenase/reductase SDR family member 11, D-beta-hydroxybutyrate dehydrogenase (mitochondrial), D-beta-hydroxybutyrate dehydrogenase (mitochondrial), and retinol dehydrogenase. The remaining two—namely, dehydrogenase/reductase SDR family member 12 and C-factor—were upregulated.

Family TC 1.A.17: This category represents transporters from the calcium-dependent chloride channel (Ca-ClC) family. Only two (calcium permeable stress-gated cation channel 1 and transmembrane channel-like protein 7) DETs were mapped to the Ca-ClC family. Both these transporters were downregulated.

In addition to the above-mentioned families, there were nine additional TC 1.A families, each represented by only one representative on the list of transporters found in this work (Table S6). Among these, five were downregulated, representing the families 1.A.79 (SID1 transmembrane family member 1), 1.A.33 (Endoplasmic reticulum chaperone BiP), 1.A.21 (Bcl-2-related ovarian killer protein), 1.A.13 (Calcium-activated chloride channel regulator 2), and 1.A.101 (Peroxisomal membrane protein 11C), respectively. The Bcl-2-related ovarian killer protein was the most downregulated of all these five transporters. The Bcl-2 family is represented by apoptosis regulator Bcl-X and its homologs [71]. Such proteins play essential roles in apoptosis, the cell suicide program necessary for development, tissue homeostasis, and protection against pathogens.

In contrast, the transporters that represented families 1.A.77 (Protein MAK16 homolog B), 1.A.75 (Piezo-type mechanosensitive ion channel component), 1.A.46 (Bestrophin-2), and 1.A.28 (Urea transporter 1) were all upregulated (Table S6). Of these, the family represented by the Piezo-type mechanosensitive ion channel component, a multi-pass, mechanically activated cation channel [72], was the most upregulated.

##### Membrane-Bound Channels (TC 1.I)

These transporters allow the transport of molecules across cells or organelle membranes [19]. Ten transporters belonging exclusively to the nuclear pore complex (NPC) family (TC 1.I.1) were found in this subclass and were identified as the most abundant transporter family (Figure 6C). Proteins that belong to the NPC family aid in the exchange of molecules between the interior of the nucleus and the cytoplasm [73]. Serine/threonine-protein kinase OSR1 (Oxidative stress-responsive 1 protein) was the only transporter of this family that was upregulated, whereas all other nine transcripts were downregulated. OSR1 is known to bind and phosphorylate PAK1 (Serine/threonine-protein kinase PAK 1) and interacts with chloride channel proteins (*SLC12A6* isoform 2, *SLC12A1*, and *SLC12A2* (*NKCC1*)), thereby initiating the cellular response to environmental stress [74]. In contrast, the downregulated transcripts belonging to this (TC 1.I.1) family comprise a polycomb protein (*EED*), two unconventional myosins (*Myo1C* and *Myo18a*), serine/threonine-protein kinase 16 (*STK16*), activating molecule in BECN1-regulated autophagy protein 1 (*AMBRA1*), mitogen-activated protein kinase kinase kinase kinase 3 (*MAP4K3*), ATP-dependent RNA helicase (*DDX3X*), cyclin-dependent kinase 9 (*CDK9*), and *MAPk*-*Ak2*). Among these, *MAPk*-*Ak2* was the most downregulated. *MAPk*-*Ak2* is a substrate of the p38 MAPK that is responsible for the signaling events affecting several cellular processes such as inflammation, division and differentiation, apoptosis, and motility in response to a variety of extracellular stimuli [75]. p38 MAPK is generally regulated by alterations in environmental osmolarity by dual tyrosine/threonine phosphorylation [76] mediated by dual specificity mitogen-activated protein kinase kinase 3 (MAPKK3 or MEK-3) and dual specificity mitogen-activated protein kinase kinase 6 (MAPKK6 or MEK-6) [77,78].

##### Miscellaneous Channels/Pore Families

In addition to TC 1.A and 1.I, two proteins each from subclass vesicle fusion pores (TC 1.F) and the non-envelope virus penetration complex (1.P) were seen among the list of DETs (Table S6). Both the TC 1.F transporters were downregulated and represented the synaptosomal vesicle fusion pores family (TC 1.F.1). Family 1.F.1 transporters are the power engines that are known to bring the membranes together [79]. The two transporters were annotated as protein ROP and vacuolar protein sorting-associated protein 45, respectively. On the other hand, one of the DETs from subclass 1.P representing mitochondrial protein tumorous imaginal discs (TID58) was highly upregulated, whereas another mitochondrial chaperone protein dnaJ 1 (*ATJ1*) was downregulated. Currently, there is only one family, the polyoma virus SV40 ER penetration channels (VPEC) (TC 1.P.1), known in this subclass. It is reported that a non-enveloped virus stimulates its own membrane translocation by initiating the release and recruitment of essential transport factors to the endoplasmic reticulum (ER) membrane [80]. The other under-represented transporter families from channels/pores (TC 1) were the Pro-Pro-Glu actinobacterial outer membrane porin (PPE) (TC 1.B.94), aerolysin channel-forming toxin (aerolysin) (TC 1.C.4), and the membrane contact site (MCS) (TC 1.R.1) families, respectively. Each of these three families were represented by a single member. Among these three, only the transcript representing TC 1.B.94 was downregulated (Table S6).

#### 3.6.4. Incompletely Characterized Transport Systems (TC 9)

This class consists of transporters that are unclassified and comprises three main subclasses (TC 9.A, 9.B, and 9.C) in the TCDB classification system. A total of 27/162 DE transcripts (that encode transporters) shared between subclasses 9.A (12) and 9.B (15) were identified from this class of transporters (Table 5). No transporter from subclass TC 9.C was found in the list of DETs identified in this work.

##### Recognized Transporters of Unknown Biochemical Mechanism (TC 9.A)

This subclass represents proteins that are recognized as transporters, but their mechanism of action is unknown [19]. TC 9.A is divided into at least 77 different families, although only five such families were identified in the meta-analysis.

Family TC 9.A.3: The members of this family represent the sorting nexin 27 (*SNX27*)-retromer assembly apparatus (RetromerAA) of transporters. Four transporters were identified in this family, and all of them were downregulated. The PDZ domain of SNX27 helps in the recycling of internalized transmembrane proteins from endosomes to the plasma membrane by linking PDZ-dependent cargo identification to retromer-mediated transport [19].

Family TC 9.A.63: The second-most abundant category with three members identified in this group represented the retromer-dependent vacuolar protein sorting (R-VPS) family. Both of these components are necessary for maintaining the vacuole membrane organization [81]. All three transporters from this family were also downregulated.

Two DETs were also found in the autophagy-related phagophore-formation transporter (APT) family (TC 9.A.15) (Table S6). One of them was upregulated (serine/threonine-protein kinase tousled-like 2: *TLK2*) by more than four-fold, while the other one—namely, lactoylglutathione lyase (*GLO1*)—was downregulated. Autophagy-related genes are known to play essential roles in the development and stress responses in plants [82]. In addition to these, three more 9.A families with a single member in each were found. These include the mitochondrial cholesterol/porphyrin/5-aminolevulinic acid uptake translocator protein (TSPO) family (TC 9.A.24), Ca2+-dependent phospholipid scramblase (Scramblase) (TC 9.A.36), and small nuclear RNA exporter (snRNA-E) (TC 9.A.60) families, respectively. All the three transcripts representing these families were downregulated; however, the transcript that potentially codes for the mitochondrial translocator protein (TSPO) was the most downregulated.

##### Putative Transport Proteins (TC 9.B)

This category consists of proteins for which a transport function has been proposed; however, there is no information to back this suggestion [19]. The proteins in this subclass could be assigned to a well-defined class when their role in transport is verified, else they will be removed from the TCDB. Currently, with 408 different families, TC 9.B is the largest subclass in the TC (date accessed: September 22, 2020) classification system. We found 15 DETs distributed in 11 different TC 9.B families.

Family TC 9.B.87: This group represents the selenoprotein P receptor (SelP-Receptor) family of putative transporters. SelP comprises most of the selenium in blood plasma, and it is used by various organs (e.g., kidneys, brain, and testes) as a selenium source for the biosynthesis of selenoprotein [19]. SelP homologs are implicated in diverse functional roles, including the regulation of ion transporters. Five DETs were identified in this family, and all of them were downregulated. Among these, Cubilin, a 460-kDa endocytic receptor, was the most downregulated transcript. Cubilin is known to be involved in several essential functions that include the absorption of vitamin B12 in the intestines, catabolism of apolipoprotein A-I, and, in general, renal protein reabsorption [83].

Additional families in which at least one DET was found are TCs 9.B.105, 9.B.135, 9.B.142, 9.B.198, 9.B.208, 9.B.229, 9.B.265, 9.B.278, 9.B.311, and 9.B.371 (Table S6). All the transcripts representing each individual family were downregulated, except in the case of the transcript (retinoic acid receptor: *RXR*) found in the TC 9.B.208 (vitamin D3 receptor (VDR) family), which was upregulated. RXR is a ligand-dependent transcription factor that may play a role in the retinoic acid response pathway [84].

#### 3.6.5. Electrochemical Potential-Driven Transporters (TC 2)

Additionally known as secondary carrier-type facilitators, TC 2 transporters use a carrier-mediated process to catalyze uniport, antiport, or symport systems [19]. Among all the abundant transporter classes, only 16/162 (9.87%) DE transporters were found in this class. This class comprises four subclasses (TCs 2.A-D); however, only DETs belonging to subclass 2.A were observed (Table 5). TC 2.A is further divided into 133 families, of which only eight were present.

Family TC 2.A.1: Proteins of this ancient, huge, and diverse family represent the major facilitator superfamily (MFS) and comprise millions of sequenced proteins [19]. Members of this superfamily catalyze uniport, symport, and/or antiport. DETs that represented glucose-6-phosphate exchanger (*SLC37A2*) and facilitated trehalose transporter Tret1-2 homolog (*TRET1-2*), putative inorganic phosphate cotransporter (Picot), and solute carrier family 49-member 4 homolog (*SLC49A4*) were identified in this family. Among these, only *SLC37A2* was upregulated, whereas the other three were downregulated.

Family TC 2.A.29: Similar to TC 2.A.1, only four DETs that encode for mitochondrial coenzyme A transporter (*SLC25A42*), mitochondrial ornithine transporter 1 (*SLC25A15*), Graves’ disease carrier protein homolog (*SLC25A16*), and mitochondrial glycine transporter (*SLC25A38*) were found in this family. Among these, only *SLC25A38* was downregulated.

Family TC 2.A.7: Only three DETs—namely, adenosine 3’-phospho 5’-phosphosulfate transporter 2 (*SLC35B3*), acid sphingomyelinase-like phosphodiesterase 3b (*SMPDL3B*), and solute carrier family 35 member F5 (*SLC35F5*)—were assigned to this drug/metabolite transporter (DMT) superfamily. All the three transcripts representing this superfamily were downregulated by more than four-fold. In general, most of the DMT superfamily members are nucleotide–sugar transporters that are involved in the endoplasmic reticulum and Golgi of eukaryotic cells [85].

Other categories of subclass TC 2.A included the zinc (Zn^2+^)-iron (Fe^2+^) permease (ZIP) (2.A.5); neurotransmitter:sodium symporter (NSS) (2.A.22); bile acid:Na+ symporter (BASS) (2.A.28); glycerol uptake (GUP) or membrane-bound acyl transferase (MBOAT) (2.A.50); and the sweet, PQ-loop, saliva;, and MtN3 (Sweet) (2.A.123) families. Only a single representative from each of these families was found in the DETs, and each of these transcripts were downregulated (Table S6).

In addition to the above-discussed transporter families that were over-represented in the DETs identified in the CMC transcriptome by the RNA-Seq meta-analysis, three additional transcripts, each belonging to different families, were also found. Among them, a transcript representing putative ferric-chelate reductase 1 homolog (*CG8399*) was the most downregulated and belonged to the eukaryotic cytochrome b561 (Cytb561) family (TC 5.B.2) of the class transmembrane electron carriers (TC 5) (Table S6). Trans-plasma membrane electron transfer is achieved by the b-type cytochromes from various families and is involved in broad cellular processes that involve two interacting redox couples physically isolated by a phospholipid bilayer—for example, the uptake of iron and redox signaling [86]. The remaining two transcripts belonged to the fatty acid transporter (FAT) (TC 4.C.1) and the putative vectorial glycosyl polymerization (VGP) (TC 4.D.1) families of the class group translocators (TC 4), respectively (Table 5 and Table S6). Members of TC 4 modify the substrate during the transport process, which involves combined chemical and vectorial reactions [19].

Overall, of the 162 DETs that encode various transporters, the majority of them were downregulated, whereas only 41 were upregulated. The analysis suggested that change in salinity triggers the differential expression of almost all the major transport classes (e.g., TC 1, TC 2, TC 3, and TC 8), as well as several unclassified transporters (TC 9). Moreover, transporters belonging to TC 8 were the most diverse and represented 25 different families. In contrast, only three transporters belonging to TC 4 and TC 5 were found. Some of these numbers might change in the future when TC 9 (unclassified) transporters are assigned to well-defined classes or removed from the transporter classification system.

## 4. Discussion

Studies have shown that gills are the most relevant tissues during osmoregulation in CMC, and to understand the expression profiles and the associated pathways, many studies have applied RNA-Seq techniques under different salinity conditions [3,9,10,16]. However, differences in the experimental conditions and the analytical pipelines used to investigate the same biological question might affect which genes are differentially expressed in each study [39]. To overcome some of these issues, a meta-analysis offers a way to systematically integrate results from multiple individual studies and remove the inconsistencies in these datasets by increasing the sample size and statistical power to detect more robust biomarkers associated with a specific condition [87]. In this work, we performed the first comprehensive meta-analysis of four separate RNA-Seq studies involving the gills of CMC under variable salinity conditions to get an overview of their gene expression profiles and, also, to identify novel or more reliable genes associated with salinity change. The integration of multiple RNA-Seq datasets in this meta-analysis identified several new potential transcripts that may serve as potential biomarkers and highlights that a broad variety of transporters are associated with salinity change.

Our meta-analysis identified 405 DETs between freshwater and high-salinity conditions. Of these, the protein argonaute-2 (*AGO2*) and mitochondrial succinate–CoA ligase (GDP-forming) subunit beta (*SUCLG2*) were encoded by the topmost down- and upregulated transcripts, respectively. Proteins belonging to the argonaute family associate with small RNAs and help in mRNA degradation, translational repression, or both [88]. In contrast, *SUCLG2* catalyzes the only step in the citric acid cycle that provides substrate-level phosphorylation to synthesize GTP from GDP [89]. While ATP is the main source of energy for many biological processes, *SUCLG2* may directly influence the anabolic functions of the citric acid cycle inside the mitochondria by supplying GTP for the GTP-dependent steps of protein synthesis [90]. One of the highly downregulated genes found in this work was tramtrack (*ttk*) (Table 3), which encodes for protein tramtrack (beta isoform) in *Drosophila melanogaster* (fruit fly). *ttk* is a transcription factor that has been implicated in several roles, including cell fate specification, cell proliferation, and cell cycle regulation. Besides these established roles, *ttk* also plays a crucial role in tracheal development [91]. Gills are one of the primary sites of ion transport and the movement of water in aquatic organisms [92]. Therefore, a series of structural and functional modifications are developed within these osmoregulatory systems so that a constant ion and osmotic homeostasis is maintained as the organism moves from low- to high-salinity conditions or vice versa. Studies have also shown that salinity also regulates the number of branchial chloride cells and their shape, as well as the expression level of several transport proteins [93]. These results are consistent with the GO and KEGG pathway enrichment analysis performed in this work, where it was found that several processes related to development and morphogenesis were over-represented (Figure 4 and Table S4).

As discussed above, a large number (162/405) of DETs were found to be transporters belonging to various classes. Transporters such as Na^+^/K^+^-ATPase and V-type ATPase are well-known biomarkers of crustacean gills [3] and have been highlighted in several studies. Although the function of these ATPases in ion transport is well-established, the role of transporters and enzymes from other classes to complete the process cannot be undermined [94]. This is supported by the fact that, in this work, a large number of differentially expressed TC 8 (accessory factors) transporters were identified. Such transporters are not directly involved in the transport of ions, but they function or form complexes with other known transporters to complete the transport [19]. Several representatives of this class were downregulated. For example, Syntenin-1 (*SDCBP*), also known as syndecan-binding protein 1, was the most downregulated transcript from the family TC 8.A.23. Syndecans are receptors for a diverse type of proteins and play essential roles in many cellular processes, such as development, maintenance, repair, cell adhesion, and migration. Reports have shown that syndecans can also regulate stretch-activated ion channels [95]. Additionally, several members of TC 8 transporters (TC 8.A.23) are indispensable for maintaining voltage-gated potassium channels [96] and regulating Na^+^/K^+^-ATPase and connexin 43 [97] or are involved in several other cellular processes, such as proliferation, migration, and survival [98]. Therefore, the identification of several accessory factors in response to salinity stress suggests that the significance of these proteins is like that of well-known transporters. These data should trigger more interest towards their functional roles in osmoregulation.

The number of TC 3 (primary active) transporters was slightly lower as compared to the accessory factors but higher than other transport classes. TC 3 transporters help in the active transport of solutes against a concentration gradient that is derived by a primary source of energy [19]. For example, some of these transporters (TC 3.A) carry out the active uptake or removal of solutes by the hydrolysis of a diphosphate bond of inorganic pyrophosphate, ATP, or other nucleoside triphosphates [19]. In contrast, other members of this class represent transport systems that are energized by the exothermic flow of electrons and consists of proteins (TC 3.D) that facilitate the transport of solutes from a reduced to an oxidized substrate [19]. Na^+^/K^+^-ATPase (TC 3.A.3) and V-type ATPase (TC 3.A.2) are two well-known examples of this class. Studies have shown that Na^+^/K^+^-ATPase is an essential component for low-salinity adaptation in blue crab (*Callinectes sapidus*) [99]. Similarly, V-ATPases (large, multi-subunit proton pumps) are another very important class of transporters that are involved in transporting hydrogen ions in exchange for energy in the form of ATP [20]. In *Drosophila*, there are 14 subunits of V-ATPase encoded by at least 33 genes [20]. Some of these subunits also have shown splice variants. Previous studies on the gills of mitten crabs have either reported no DE V-ATPase subunits [10] or highlighted only few subunits [3,9,16]. As discussed above, we identified several DE subunits of V-ATPase, of which some exhibited a similar trend in their expression, whereas, for others, an inconsistent expression pattern was observed. In spite of the well-recognized role of V-ATPase, it is worth mentioning that its function depends on *RNASEK*, which closely associates with this enzyme [100]. Decline in the expression of *RNASEK* changes the localization of various V-ATPase subunits and effects the expression levels of some of its subunits. Therefore, to better understand a complete mechanism such as salinity change, it is of importance to identify the interacting partners (or co-expressed genes) of the well-known biomarkers and explore the consequent effects on the changing condition.

TC 1 proteins aid in transport through an energy-independent (facilitated diffusion) process in which the substrate moves across the channel or pore without the coupling of the translocation step to another chemical or vectorial process [101]. Some of these channels (e.g., TC 1.A) are found in all organisms and catalyze the transport of solutes by an energy-independent process by passage through a channel or pore with no indication of a carrier-mediated mechanism [19]. These data suggest that such transporters have widespread and conserved physiological functions [102]. The transport of ions through channels, especially the ingress of calcium ions into the cytosol, serves as an indication for environmental responses in eukaryotic organisms [103]. In fact, in plants, a swift upsurge in the cytosolic-free Ca^2+^ concentration is one of the initial events observed after osmotic stress [104]. Members of the Ca-ClC family are cation channels that are permeable to calcium and gated by physical signals such as osmotic stress [103]. In animals, such channels are necessary for normal electrolyte and fluid secretion, olfactory perception, and neuronal, as well as smooth muscle, excitability [105]. Similarly, mechanosensitive channels are transmembrane proteins with pores that mediate the flow of ions or osmolytes across membranes in response to mechanical stimuli. In animals, they are essential for somatosensory perception, whereas, in plants, in addition to their sensory and regulatory roles, they are indispensable for responses to osmotic shock [106].

In our meta-analysis, we observed that the number of secondary carriers is much lower as compared to other transporter classes (Figure 6). Additionally, only one subclass (TC 2.A) was found in this work and includes transporters that employ a carrier-mediated process to catalyze uniport (a single species is transported), antiport (two or more species are transported in opposite directions), and/or symport (two or more species are transported together in the same direction) [19]. One of the most widely distributed families of this subclass among all living organisms is the major facilitator superfamily (MFS), also known as the uniporter–symporter–antiporter family [107]. Such transporters are single-polypeptide secondary carriers that can transport only small solutes in response to chemiosmotic ion gradients. MFS transporters utilize the ionic (H^+^) gradient across the membrane as the source of energy, a mechanism well-conserved among living organisms [108]. Another important family within secondary carriers is the mitochondrial carrier (MC) family (the human SLC25 family), which, in general, prefers the antiport (exchange of a solute for some other) system [19]. Individuals of this family participate in the transport of keto acids, amino acids, nucleotides, inorganic ions, and cofactors across the mitochondrial inner membrane. SLC25 transporters serve as a bridge between metabolic reactions of the cytosol and mitochondrial matrix (or various cell compartments) by catalyzing the transfer of diverse solutes across the membrane. They also participate in numerous crucial metabolic pathways, such as oxidative phosphorylation, the citric acid cycle, Ca^2+^-cell signaling, and cell death [109]. Although only a limited number of DE transporters belonging to TC 2 were identified, the importance of this transporter class during changes in the environmental conditions such as salinity cannot be under-represented.

Several transporters of unknown classification were also found distributed between two subclasses (TC 9.A and 9.B) of this group. The proteins of TC 9.A include transporters with unknown biochemical mechanisms; however, their role in transport has been established [19]. In contrast, TC 9.B consists of putative transporters with no evidence of a transport-related function. Once their role in transport is established, they will be assigned to a relevant group or removed from the transport classification system if proven otherwise. In our network analysis, many of the TC 9 transporters are grouped together with TC 8 transporters (Figure 5), suggesting that some of these may either be assigned to the TC 8 category or, probably, they might carry out the transport-related functions in coordination.

## 5. Conclusions

Overall, in this meta-analysis of RNA-Seq data, we successfully identified a number of transcripts whose expressions were apparently altered in the gills of Chinese mitten crabs in response to ambient changes in the salinity conditions. Of these DETs, a large number were found to be transporters from diverse families, again highlighting the significance and greater contribution of the transportome in osmotic regulation. Coordinated responses induced by salinity changes triggered several processes related to cell development, differentiation, motility, and protein synthesis, including others, as some of the most enriched processes. The processes and the DETs identified from our meta-analysis will serve as more robust candidates for understanding the mechanism of osmotic regulation in *E. sinensis* as compared to the genes and pathways reported from individual studies. This study, in addition to being the first meta-analysis of CMC in response to salinity, also represents the first such study that provides a focused overview of various transporters involved in salinity change. The various types of transporters highlighted in this meta-analysis can provide significant clues for experimental scientists to design future experiments to get a better understanding of the adaptation to salinity change and, consequently, the underlying mechanisms of osmotic regulation at varying salinity conditions in Chinese mitten crabs.

## Figures and Tables

**Table 1:** Summary of the datasets and the number of trimmed reads retained postfiltering.

Dataset	BioProject Accession	Type *	#Raw Reads	#Trimmed Reads	Reads Retained Postfiltering (%)	Reference
DS1	PRJNA481259	PE	49,648,084	47,331,397	95.33	[3]
DS2	PRJNA488907		17,817,553,2	14,353,948,4	80.42	[16]
DS3	PRJNA80779		64,212,034	60,050,212	93.51	[10]
DS4	PRJNA508867	SE	75,845,511	75,292,415	99.28	[9]

* PE = Paired-end. SE = Single-end.

**Table 2:** Top 20 highly correlated differentially expressed transcripts (DETs). P1 and P2 represent the first and the second proteins in a correlated pair. Direction of the arrow represents whether the representative transcript is downregulated (↓) or upregulated (↑).

P1	P1 Description	P2	P2 Description	Effect	Cor	Corrected *p*-Value
Q9D4P0	ADP-ribosylation factor-like protein 5B	B2D0J5	Venom carboxylesterase-6	↓	1	9.4355 × 10^−7^
Q7ZV80	Survival of motor neuron-related-splicing factor 30	B0WTN3	Eukaryotic translation initiation factor 3 subunit M	↓	0.99	1.2953 × 10^−6^
Q04164	Putative epidermal cell surface receptor	Q9UQC9	Calcium-activated chloride channel regulator 2	↓	0.99	1.5143 × 10^−6^
Q8C0K5	Graves disease carrier protein homolog (GDC) (Mitochondrial solute carrier protein homolog)	Q13336	Urea transporter 1	↑	0.99	3.4321 × 10^−6^
Q5REW1	Iodotyrosine deiodinase 1 (IYD-1)	Q5I7G2	Retinoic acid receptor RXR	↑	0.99	3.4321 × 10^−6^
Q9Y535	DNA-directed RNA polymerase III subunit RPC8	Q5VWG9	Transcription initiation factor TFIID subunit 3	↓	0.99	3.4321 × 10^−6^
Q04164	Putative epidermal cell surface receptor	Q27421	Protein outspread	↓	0.99	3.4321 × 10^−6^
Q8WXS8	A disintegrin and metalloproteinase with thrombospondin motifs 14	O16011	Protein muscleblind	↓	0.99	3.4321 × 10^−6^
O74503	Upstream activation factor subunit spp27	Q53CF6	Cytochrome c oxidase subunit 7A1, mitochondrial	↑	0.99	3.7031 × 10^−6^
Q4R3Y4	Long-chain fatty acid transport protein 4 (FATP-4)	Q7ZV80	Survival of motor neuron-related-splicing factor 30	↓	0.99	3.9073 × 10^−6^
Q68HB4	Profilin	P56616	Ubiquitin-conjugating enzyme E2 C	↓	0.99	6.1125 × 10^−6^
Q4R3Y4	Long-chain fatty acid transport protein 4(FATP-4)	B0WTN3	Eukaryotic translation initiation factor 3 subunit M	↓	0.99	6.1125 × 10^−6^
Q86WZ6	Zinc finger protein 227	Q8C0K5	Graves disease carrier protein homolog	↑	0.99	7.7076 × 10^−6^
P53590	Succinate–CoA ligase (GDP-forming) subunit beta, mitochondrial	P21158	C-factor	↑	0.99	7.7076 × 10^−6^
Q9D4P0	ADP-ribosylation factor-like protein 5B	P82968	Four-domain proteases inhibitor	↓	0.99	7.7076 × 10^−6^
Q04833	Low-density lipoprotein receptor-related protein	P82968	Four-domain proteases inhibitor	↓	0.99	8.8569 × 10^−6^
Q9VB68	Serine protease grass	Q5VWG9	Transcription initiation factor TFIID subunit 3	↓	0.99	8.8569 × 10^−6^
Q8K0U4	Heat shock 70-kDa protein 12A	Q8N539	Fibrinogen C domain-containing protein 1	↓	0.99	1.2159 × 10^−5^
A6QP05	Dehydrogenase/reductase SDR family member 12	Q9CY58	Plasminogen activator inhibitor 1 RNA-binding protein	↑	0.99	1.5922 × 10^−5^
P29844	Endoplasmic reticulum chaperone BiP	Q91V92	ATP-citrate synthase	↓	0.99	1.6280 × 10^−5^

**Table 3:** List of six transcripts and their annotations (based on BLASTp) that were commonly identified in the individual datasets and the Fisher method. Direction of the arrow represents whether the representative transcript is downregulated (↓) or upregulated (↑).

UniProt ID	Gene Name	Organism	Protein Name	Gene Ontology (Biological Process)	Effect
P17789	*ttk*	*Drosophila melanogaster* (Fruit fly)	Protein tramtrack, beta isoform	branch fusion, open tracheal system (GO:0035147)	↓
P53590	*SUCLG2*	*Sus scrofa* (Pig)	Succinate–CoA ligase (GDP-forming) subunit beta, mitochondrial	succinyl–CoA metabolic process (GO:0006104)	↑
Q28BZ2	*Hint3*	*Xenopus tropicalis* (Western clawed frog)	Histidine triad nucleotide-binding protein 3	NA	↑
Q5U2Q3	NA	*Rattus norvegicus* (Rat)	Ester hydrolase C11orf54 homolog	NA	↑
Q6DIS8	*Lzts2*	*Xenopus tropicalis* (Western clawed frog)	Leucine zipper putative tumor suppressor 2 homolog	microtubule severing (GO:0051013)	↓
Q8BTN6	*Leng9*	*Mus musculus* (Mouse)	Leukocyte receptor cluster member 9	NA	↓

**Table 4:** Enriched KEGG pathways detected in the DETs identified by the RNA-Seq meta-analysis of Chinese mitten crab (CMC) gills under salinity.

Pathway Name	Pathway ID	No. of Input Genes	Total No. of Genes	Corrected *p*-Value
Metabolic pathways	dme01100	52	1111	0.00001
Apoptosis—fly	dme04214	9	63	0.00034
Phagosome	dme04145	10	89	0.00067
ECM-receptor interaction	dme04512	4	12	0.00316
Oxidative phosphorylation	dme00190	11	144	0.00471
Endocytosis	dme04144	10	122	0.00497
Glycerophospholipid metabolism	dme00564	6	63	0.02182
Pyruvate metabolism	dme00620	5	46	0.02654
Sphingolipid metabolism	dme00600	4	28	0.02680
Spliceosome	dme03040	8	128	0.04231
Glycolysis/Gluconeogenesis	dme00010	5	55	0.04283
Protein processing in endoplasmic reticulum	dme04141	8	133	0.04868

**Table 5:** List of the most abundant transporter classes, including their representative subclasses and families. Numbers in parenthesis indicate the total number of differentially expressed transcripts assigned to that specific class, subclass, and family. Direction of the arrow represents whether the representative transcript is downregulated (↓) or upregulated (↑).

Class	Class Description	Subclass	Subclass Description	Family	Family Description	Representative Transcript	Effect
TC 8 (45)	Accessory Factors Involved in Transport	TC 8.A (43)	Auxiliary transport proteins	TC 8.A.24 (6)	The Ezrin/Radixin/Moesin-binding Phosphoprotein 50 (EBP50) family	Syntenin-1	↓
				TC 8.A.23 (4)	The Basigin family	Tyrosine-protein kinase Abl	↓
				TC 8.A.104 (4)	The 5’-AMP-activated protein kinase (AMPK) family	Serine/threonine-protein kinase pim-3	↓
		TC 8.B (2)	Ribosomally synthesized protein/peptide toxins/agonists that target channels and carriers	TC 8.B.14 (2)	The Sea Anemone Peptide Toxin, Class 1 (BgK) family	Matrix metalloproteinase-24	↓
TC 3 (37)	Primary Active Transporters	TC 3.A (33)	P-P-bond hydrolysis-driven transporters	TC 3.A.2 (6)	The H+- or Na+-translocating F-type, V-type and A-type ATPase (F-ATPase) superfamily	V-type proton ATPase subunit a1	↓
				TC 3.A.16 (6)	The Endoplasmic Reticular Retrotranslocon (ER-RT) family	26S proteasome regulatory subunit 6A	↑
				TC 3.A.5 (4)	The General Secretory Pathway (Sec) family	Putative U5 small nuclear ribonucleoprotein 200-kDa helicase	↓
		TC 3.D (4)	Oxidoreduction-driven transporters	TC 3.D.1 (3)	The H+ or Na+-translocating NADH Dehydrogenase (NDH) family	NADH dehydrogenase (ubiquinone) iron-sulfur protein 8, mitochondrial	↑
				TC 3.D.4 (1)	The Proton-translocating Cytochrome Oxidase (COX) Superfamily	Cytochrome c oxidase subunit 7A1, mitochondrial	↑
TC 1 (34)	Channels/Pores	TC 1.A (17)	α-Type Channels	TC 1.A.115 (6)	The Nonselective Cation Channel-2 (NSCC2) family	Dehydrogenase/reductase SDR family member 12	↑
				TC 1.A.17 (2)	The Calcium-dependent Chloride Channel (Ca-ClC) family	Transmembrane channel-like protein 7	↓
		TC 1.I (10)	Membrane-bounded Channels	TC 1.I.1 (10)	The Nuclear Pore Complex (NPC) family	MAP kinase-activated protein kinase 2	↓
TC 9 (27)	Incompletely Characterized Transport Systems	TC 9.A (12)	Recognized Transporters of Unknown Biochemical Mechanism	TC 9.A.3 (4)	The Sorting Nexin27 (SNX27)-Retromer Assembly Apparatus	Ras-related protein Rap-1b	↓
				TC 9.A.63 (3)	The Retromer-dependent Vacuolar Protein Sorting (R-VPS) family	Cell division control protein 42 homolog	↓
		TC 9.B (15)	Putative uncharacterized transport proteins.	TC 9.B.87 (5)	The Selenoprotein P Receptor (SelP-Receptor) family	Cubilin	↓
TC 2 (16)	Electrochemical Potential-driven Transporters	TC 2.A (16)	Porters (uniporters, symporters, antiporters)	TC 2.A.1 (4)	The Major Facilitator Superfamily (MFS)	Solute carrier family 49 member 4 homolog	↓
				TC 2.A.29 (4)	The Mitochondrial Carrier (MC) family	Mitochondrial coenzyme A transporter SLC25A42	↑
				TC 2.A.7 (3)	The Drug/Metabolite Transporter (DMT) Superfamily	Solute carrier family 35 member F5	↓
TC 4 (2)	Group Translocators	TC 4.C (1)	Acyl CoA ligase-coupled transporters	TC 4.C.1 (1)	The Fatty Acid Transporter (FAT) Family	Long-chain fatty acid transport protein 4	↓
		TC 4.D (1)	Polysaccharide Synthase/Exporters	TC 4.D.1 (1)	The Putative Vectorial Glycosyl Polymerization (VGP) Family	Beta-1,4-mannosyltransferase egh	↓
TC 5 (1)	Transmembrane Electron Carriers	TC 5.B (1)	Transmembrane 1-Electron Transfer Carriers	TC 5.B.2 (1)	The Eukaryotic Cytochrome b561 (Cytb561) Family	Putative ferric-chelate reductase 1 homolog	↓

## Data Availability

The data presented in this study are available within the article. If required, any additional data is available on request from the authors.

## Supplementary Materials

The supplementary materials can be found at https://www.mdpi.com/2079-7737/10/1/39/s1: Figure S1: Assembly statistics showing (A) the percentage of orthologs identified from the BUSCO search against the Arthropoda lineage; (B) annotation of transcripts against Pfam, UniProt, Crabdb, and KEGG databases using BLASTp; and (C) total number of transcripts with open reading frames in each species identified using the TransDecoder. For BLASTp, transcripts with the topmost hits at 30% coverage and percent (%) identity at an evalue cutoff of 1e-5 were selected. For Pfam, the numbers represent the unique number of transcripts that are mapped to this database. Figure S2: The distribution of various COG categories assigned to the transcripts originating from each individual study. The COG categories are Function unknown (S); Signal transduction mechanisms (T); Posttranslational modification, protein turnover, and chaperones (O); Translation, ribosomal structure, and biogenesis (J); Transcription (K); RNA processing and modification (A); Intracellular trafficking, secretion, and vesicular transport (U); Carbohydrate transport and metabolism (G); Energy production and conversion (C); Amino acid transport and metabolism (E); Cytoskeleton (Z); Lipid transport and metabolism (I); Inorganic ion transport and metabolism (P); Replication, recombination, and repair (L); Cell cycle control, cell division, and chromosome partitioning (D); Secondary metabolites biosynthesis, transport, and catabolism (Q); Chromatin structure and dynamics (B); Nucleotide transport and metabolism (F); Coenzyme transport and metabolism (H); Cell wall/membrane/envelope biogenesis (M); Extracellular structures (W); Defense mechanisms (V); Cell Motility (N); and Nuclear structure (Y). Figure S3: Interaction network of differentially expressed transcripts. Red, green, and cyan nodes represent up- (square), downregulated (circle), and STRING-predicted (diamonds) genes, respectively. Nodes in purple indicate the hub genes labeled with orange text. Table S1: Assembly statistics of each dataset. Table S2: List of additional DETs identified by the meta-analysis. (↓) indicates that the representative transcript is downregulated. Table S3: List of DETs (2-Fold change) identified by the RNA-Seq meta-analysis. Annotations for each transcript are based on the BLASTp analysis against the UniProt database. Direction of the arrow represents whether the representative transcript is downregulated (↓) or upregulated (↑). Table S4: List of enriched GO categories in terms of the biological process (BP), molecular function (MF), and cellular component (CC). Table S5: Enrichment analysis of each individual cluster identified in the interaction network. Clusters with <10 nodes were excluded from the enrichment analysis. Table S6: List of DETs that were assigned to a transporter family based on the BLASTp analysis against the TCDB. Direction of the arrow represents whether the representative transcript is downregulated (↓) or upregulated (↑).
