# Supplementary material for: Role of Transportome in the Gills of Chinese Mitten Crabs in Response to Salinity Change: A Meta-Analysis of RNA-Seq Datasets
Source: Biology (Basel). 2021 Jan 8;10(1):39. doi: 10.3390/biology10010039 (PMC7827906; doi:10.3390/biology10010039)
Supplement: Supplementary file 1 [file biology-10-00039-s001.zip › Supplementary-data/Table-S1-S2.docx]

**Table S1: Assembly statistics of each dataset.**

| **Dataset** | **#Assembled bases** | **#Contigs** | **Median contig length** | **Average contig length** | **N50** | **Reads mapping back to the assembly (%)** |
| --- | --- | --- | --- | --- | --- | --- |
| DS1 | 223,783,322 | 285,396 | 413 | 784.12 | 1302 | 95.44 |
| DS2 | 352,474,313 | 463,905 | 424 | 759.8 | 1154 | 96.53 |
| DS3 | 646,104,65 | 72,671 | 488 | 889.08 | 1526 | 94.58 |
| DS4 | 176,822,208 | 280,314 | 334 | 630.8 | 974 | 97.54 |

**Table S2: List of additional DETs identified by meta-analysis. (↓) indicates that the representative transcript is down-regulated.**

| **Uniprot ID** | **Gene Name** | **Organism** | **Protein Name** | **GO (BP)** | **Effect** |
| --- | --- | --- | --- | --- | --- |
| Q26250 | NA | *Nephrops norvegicus*  (Norway lobster) | V-type proton ATPase 16 kDa proteolipid subunit | NA | **↓** |
| Q7Z699 | *SPRED1* | *Homo sapiens* (Human) | Sprouty-related, EVH1 domain-containing protein 1 | Inactivation of MAPK activity | **↓** |
| Q9NHD5 | *san span, CG12352* | *Drosophila melanogaster*  (Fruit fly) | Probable N-acetyltransferase san | Histone acetylation | **↓** |
| Q9UHV9 | *PFDN2 PFD2, HSPC231* | *Homo sapiens* (Human) | Prefoldin subunit 2 | Positive regulation of cytoskeleton organization | **↓** |
