# Supplementary figures and images for: Role of Transportome in the Gills of Chinese Mitten Crabs in Response to Salinity Change: A Meta-Analysis of RNA-Seq Datasets
Source: Biology (Basel). 2021 Jan 8;10(1):39. doi: 10.3390/biology10010039 (PMC7827906; doi:10.3390/biology10010039)

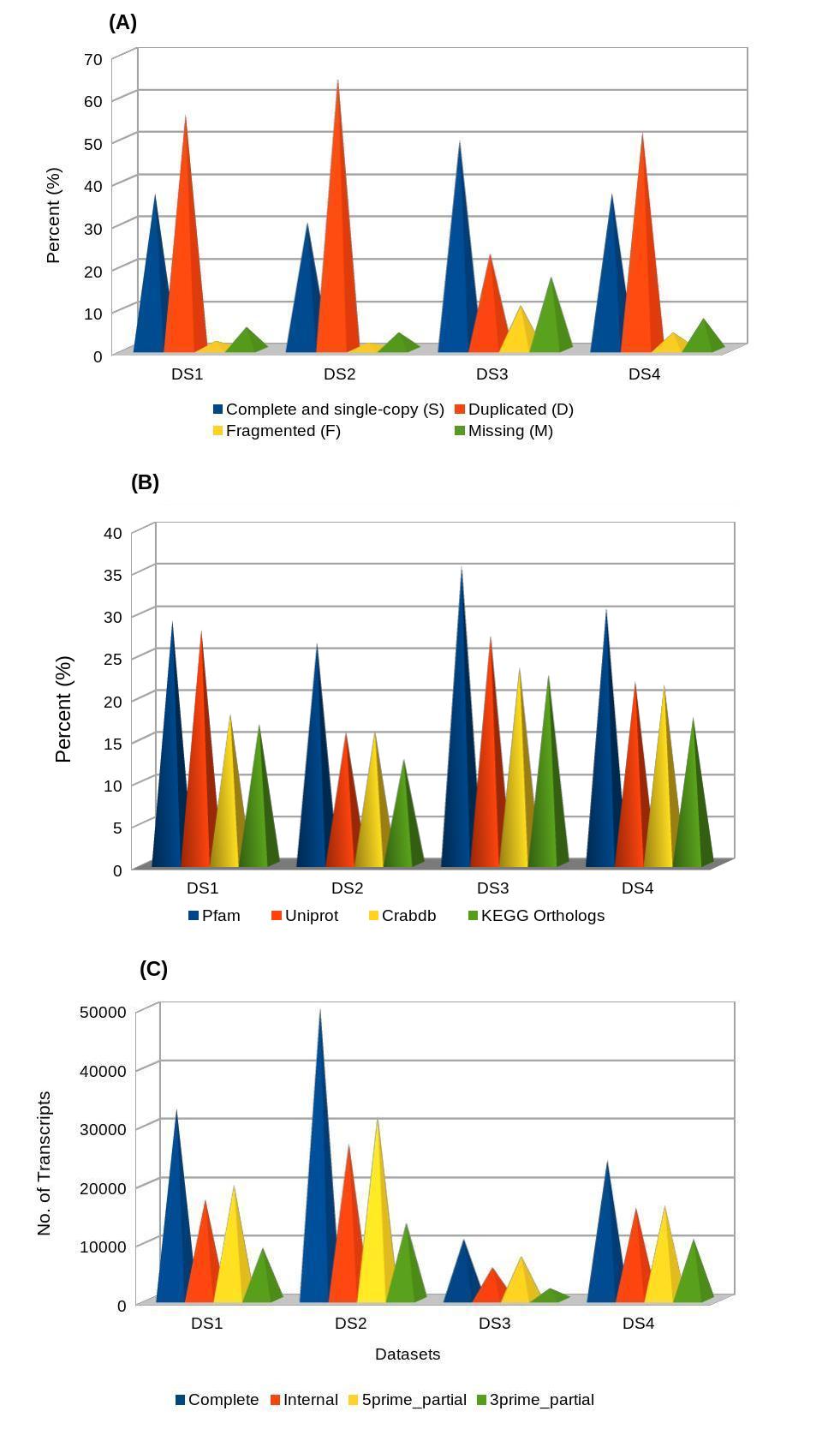

Supplement: Supplementary file 1 [file biology-10-00039-s001.zip › Supplementary-data/Figure-S1.jpg]

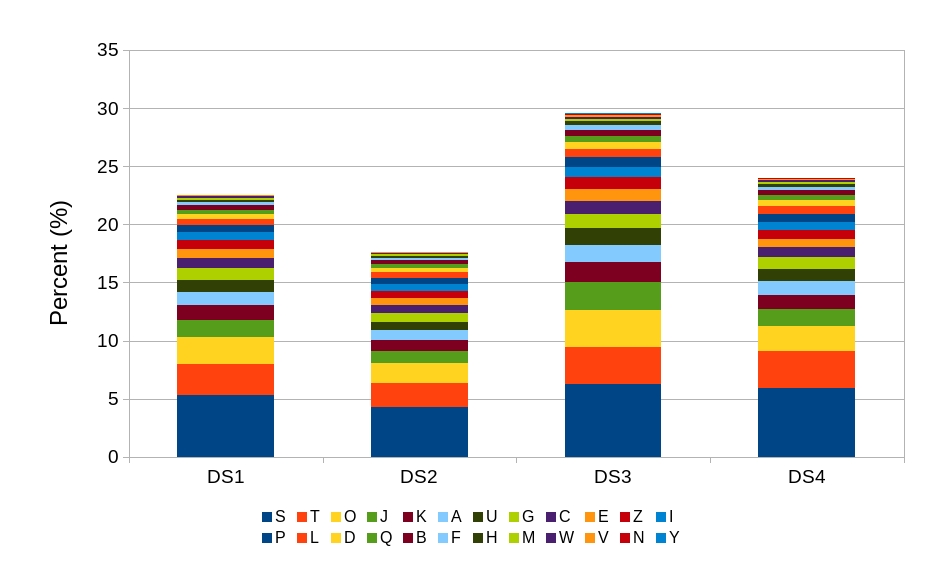

Supplement: Supplementary file 1 [file biology-10-00039-s001.zip › Supplementary-data/Figure-S2.jpg]

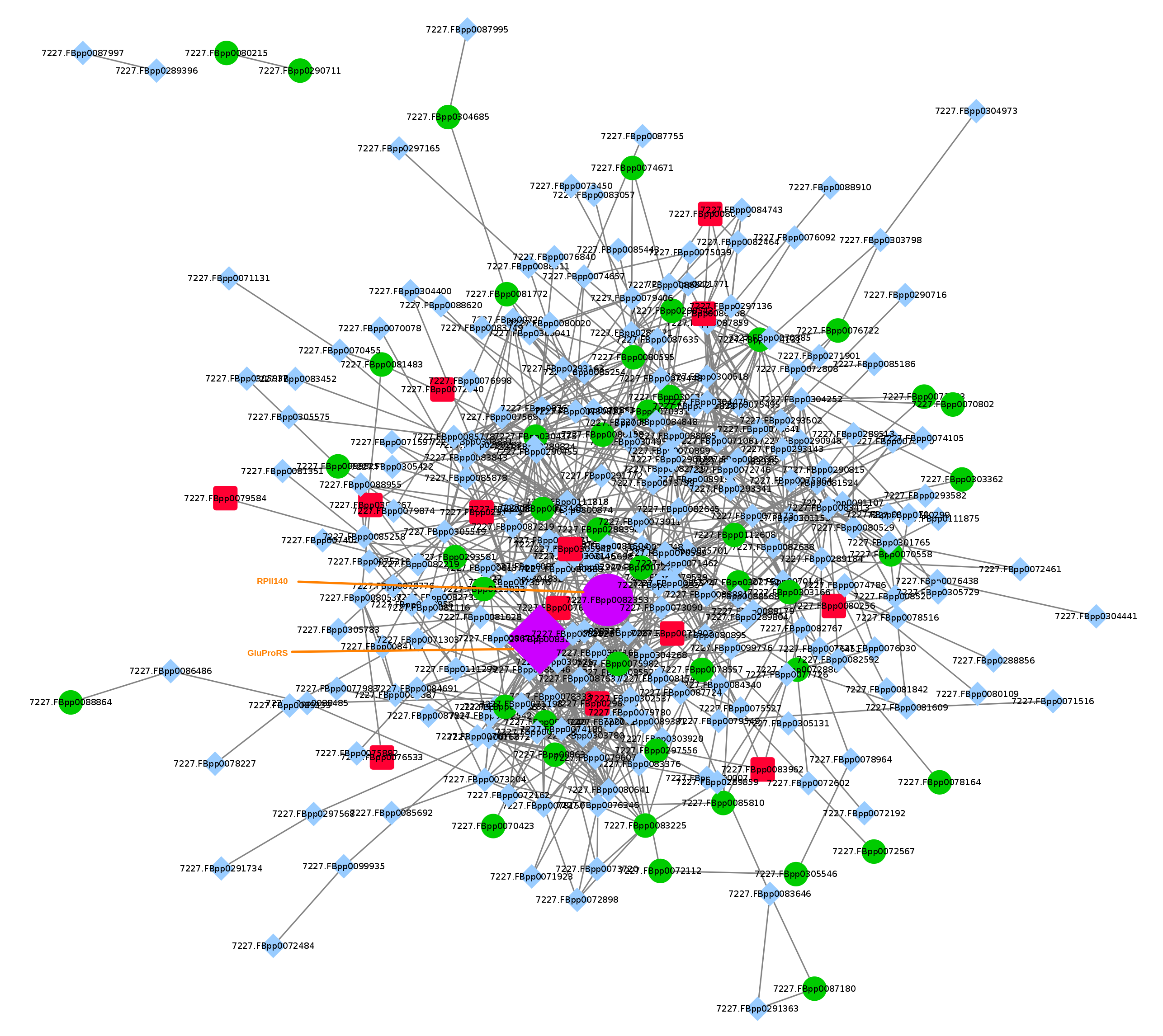

Supplement: Supplementary file 1 [file biology-10-00039-s001.zip › Supplementary-data/Figure-S3.png]
